# Advances and Emerging Techniques in Transarterial Chemoembolization for Hepatocellular Carcinoma

**DOI:** 10.3390/cancers18030514

**Published:** 2026-02-04

**Authors:** Hunter L. Gazda, Phuoc-Hanh D. Le, Ankit Patel, Arjun Jha, Mina S. Makary

**Affiliations:** 1 Division of Interventional Radiology, Department of Radiology, The Ohio State University Wexner Medical Center, 395 W 12th Ave, 4th Floor Faculty Office Tower, Columbus, OH 43210, USA; gazd05@osumc.edu; 2 College of Medicine, The Ohio State University, 1645 Neil Avenue, Columbus, OH 43210, USA; phuoc-hanh.le@osumc.edu (P.-H.D.L.); arjun.jha@osumc.edu (A.J.); 3 College of Medicine, Northeast Ohio Medical University, 4209 St. Rt. 44, Rootstown, OH 44272, USA; apatel18@neomed.edu

**Keywords:** hepatocellular carcinoma, transarterial chemoembolization, superselective, immunotherapy, ablation, radiation, radiomics

## Abstract

HCC is the third leading cause of cancer-related death, which carries a low 5-year survival rate, in part due to many patients presenting in late-stage disease. Surgical resection and liver transplantation are first-line treatments for early-stage disease, offering the chance for curative resection and, in the case of transplantation, elimination of the underlying disease process. However, due to either late-stage presentation or prohibitive surgical risk, many patients are not candidates for such treatments. Locoregional therapies, including TACE, provide treatment options for these patients. TACE is a first-line treatment recommendation for intermediate-stage disease, and in certain clinical situations it is appropriate in earlier and later stages. Improved treatment efficacy and expanded recommendations for TACE are a result of advances in the procedural technique, materials, and combination therapies. Promising combination therapies employ TACE with radiation, ablation and novel immunotherapies. When combined with new prognostic models, the treatment landscape for HCC continues to change. This review will summarize advanced TACE techniques and the new TACE combination therapies, before looking ahead at prognostic models being developed and future directions with HCC treatment.

## Simple Summary

Hepatocellular carcinoma (HCC) is the third leading cause of cancer-related death and is often asymptomatic until late-stage disease when treatment options are limited. Surgical resection and transplantation remain the gold standard for early-stage disease. However, these are not options for those presenting in later stages or those with prohibitive surgical risk. Transarterial chemoembolization (TACE) is a first-line treatment recommendation for patients with intermediate-stage disease and advances are looking to expand treatment indications and improve outcomes. Enhanced TACE techniques with new materials and procedural methods have already shown promising results. Novel TACE combination therapies with radiation, ablation, and immunotherapy are looking to advance this area further. This review will highlight advances in TACE, summarize new prognostic models and discuss future directions with HCC treatment.

## 1. Introduction

HCC is the most common primary liver cancer and the third leading cause of cancer-related death overall [1,2]. Chronic liver disease and cirrhosis often predate HCC, with multiple well-proven risk factors linked to pathogenesis. It is estimated that 56% of HCC cases are attributable to the hepatitis B virus (HBV), and 20% to the hepatitis C virus (HCV) [3]. Metabolic conditions, which are increasingly prevalent, and exposure to various toxins are also linked to the development of HCC [3,4,5,6]. Prognosis is often poor, with 5-year survival rates of less than 20% [1]. Morbidity is high, with many experiencing significant chronic pain [7]. Surgical resection and transplantation are first-line treatments for early-stage disease. Liver transplant is often considered the preferred treatment given its ability to eliminate both the HCC and the underlying liver disease [8]. When the commonly used Milan criteria for liver transplantation are satisfied, 4-year survival rates approach 75% [8]. Despite increased survival rates, HCC recurrence is still between 10 and 16% following transplantation [8]. However, many patients are not candidates for transplantation or surgical resection secondary to late-stage disease on presentation or prohibitive surgical risk from underlying cirrhosis or other comorbidities [1,9]. Systemic treatments have limited roles outside of adjunct or palliative indications in late-stage disease. Locoregional therapies can offer definitive treatment for some patients or act as adjunctive treatments to downstage for liver transplantation, resection, or curative intent locoregional therapy [10,11,12].

TACE is a locoregional therapy that allows for the simultaneous embolization of a tumor’s blood supply and local delivery of chemotherapeutic agents. TACE is a first-line treatment for intermediate-stage disease, as determined by the Barcelona Clinic Liver Cancer (BCLC) staging classification [1,9,10]. In very early-stage and early-stage disease, TACE may be considered for those who are ineligible for resection, ablation or transplantation. TACE offers these patients in earlier stages an effective locoregional therapy while preserving as much liver function as possible and without precluding further treatment options. Additionally, TACE has been utilized as a bridge to maintain transplant eligibility and to downstage patients with intermediate-stage disease for transplant consideration [1]. On occasion, TACE is considered as a second-line option in advanced-stage disease for those unable to tolerate systemic therapy or as an adjunct to systemic therapy for local control [1].

With a majority of patients presenting in later stages of disease, TACE is a foundational treatment modality for HCC. Early research improved the safety and efficacy of TACE by modifying the materials and techniques of the procedure. The first version of TACE, conventional TACE (cTACE), used an emulsion of ethiodized oil and chemotherapy. Now, in the commonly used drug-eluting bead TACE (DEB-TACE), embolic microspheres coated with chemotherapeutic agents are used for chemoembolization [10]. On the technique side, selective and superselective methods have further increased treatment efficacy and decreased adverse effects. In recent studies, the utility of combination therapy with TACE was investigated. TACE coupled with ablation or radiation therapy (RT) has the potential to synergistically treat HCC. Similarly, TACE with immunotherapy has shown promising results with the increased understanding of the immune response in the tumor microenvironment [13]. At the same time, improved predictive and prognostic models are being developed to help better guide treatment decisions. With these ongoing investigations, the indications for TACE with and without various combination therapies will continually change the treatment landscape for HCC. This study aims to summarize advanced TACE techniques, analyze TACE combination therapies, and discuss future directions in this area of HCC treatment.

### 1.1. TACE Outcomes

Multiple studies have analyzed the survival benefit of TACE across HCC stages, with most focusing on intermediate-stage HCC. TACE in intermediate-stage HCC has overall survival (OS) rates of 1, 3, 4, and 5 years of 88.2%, 64,4%, 47.4% and 39.4%, respectively [1]. Heterogeneity among reported survival outcomes for intermediate-stage HCC is likely secondary to the significant variability of patient disease status in this group. In very early- and early-stage HCC, in patients not eligible for transplantation, 1-, 2-, and 3-year OS rates of 90.9%, 86.1%, and 80.5% have been reported following TACE therapy [1]. In a meta-analysis, TACE after surgical resection in very early- and early-stage HCC has shown increased OS and disease-free survival (DFS). Notably, in the subgroup analysis, patients without microvascular invasion had no improvement in OS and worse DFS when compared to curative resection [14]. These findings suggest TACE after resection may be beneficial in cases with microvascular invasion. Advanced-state HCC is typically treated with systemic therapy. However, some patients with advanced-stage disease are considered for combination therapy with TACE if locoregional control is needed. Sorafenib, the kinase inhibitor with anti-angiogenesis activity, is the recommended systemic therapy in this group, with various new monotherapy and combination systemic medications under investigation. TACE used in combination with Sorafenib in advanced-stage HCC has shown beneficial results with an improved median OS of 15.5 months compared to 8.3 months with systemic therapy alone [15]. Concordant results have been published at the meta-analysis level with improvement in OS and time to progression (TTP) with combination TACE and sorafenib versus TACE alone [1].

### 1.2. Transarterial Embolization and Transarterial Radio-Embolization

While TACE is frequently performed for intermediate-stage HCC, it is worth mentioning the other catheter-directed locoregional therapies of transarterial embolization (TAE) and transarterial radio-embolization (TARE). TAE, also referred to as bland embolization, uses embolic material to embolize a tumor’s arterial supply without the added chemotherapy agents. The efficacy of TACE over TAE remains controversial, with a relatively limited number of studies directly comparing the two therapies. A commonly cited randomized controlled trial (RCT) comparing TAE or TACE against symptomatic treatment for unresectable HCC was stopped early when TACE showed survival benefits over symptomatic treatment [16]. Survival probabilities at 1 and 2 years for TACE were 82% and 63% compared to 63% and 27% for the control group (*p* = 0.009) [16]. The reported 1- and 2-year survival probabilities for TAE were 75% and 50% respectively; however, the null hypothesis in the TAE group could not be tested due to the trial being stopped early [16]. A few studies have reported higher response rates for various surrogate markers following TACE over TAE. A propensity score analysis comparing TACE versus TAE in intermediate-stage HCC demonstrated an increased complete radiological response (OR = 8.5; 95% CI: 2.8–25.4) for TACE [17]. However, no difference in the overall response rate or in terms of OS, progression-free survival (PFS) and transplantation-free survival were found [17]. Importantly, there was no difference in the adverse event rates between the two groups [17]. A recent systematic review and meta-analysis also found no difference in the OS and PFS when comparing TACE versus TAE in HCC [18]. The difference in adverse events between the two groups was also not statistically significant [18]. While TACE remains the most commonly performed embolization therapy for intermediate-stage HCC, the efficacy over TAE remains controversial. Further high-level investigations are needed to elucidate if one therapy is better suited for patients with HCC.

TARE, also referred to as selective internal radiotherapy (SIRT), is a local catheter-directed therapy that administers radioactive Yttrium^90^ (Y-90) in the form of glass or resin-based microparticles [12,19]. Y-90 emits beta particles with local penetration of a few centimeters allowing for precise delivery [9]. Typically in TARE, the dose is either administered in a segmental fashion to one or two segments for radio-segmentectomy or in a lobar fashion with a lower dose [9]. The indications for TARE are largely dependent on disease stage and liver function. Notably, due to the decreased risk of ischemia secondary to the reduced dose of embolic particles, TARE is sometimes preferred in patients with decreased liver function or portal vein thrombus who may be at risk of liver failure [9]. TARE in intermediate-stage HCC has demonstrated 1- and 3-year OS of 63% and 27%, respectively. In advanced-stage HCC, 1- and 3-year OS of 37% and 13% has been reported with TARE [19]. The TRACE RCT compared TARE vs. DEB-TACE in unresectable HCC and found superior outcomes with TARE [20]. Specifically, during the interim analysis, median time to overall tumor progression was 17.1 months with TARE and 9.5 months with DEB-TACE [20]. Median OS was 30.2 months with TARE and 15.6 months with DEB-TACE [20]. On the contrary, a recent multicenter retrospective study found better outcomes with TACE compared to TARE in early- and intermediate-stage HCC [21]. In particular, the median OS in BCLC 0/A patients was longer with TACE at 60 months compared to 25 months with TARE [21]. However, the survival outcomes in the BCLC B group were not statistically significant and the overall tumor diameter was significantly larger in the TARE group [21]. Further research is required to elucidate which therapy has better outcomes, with results likely dependent on individual patient scenario.

## 2. TACE

### 2.1. cTACE and DEB-TACE

In TACE, cTACE and DEB-TACE are the two commonly described techniques [22,23,24]. In cTACE, intra-arterial administration of chemotherapy is accomplished in a lipiodol emulsion. Doxorubicin, cisplatin, and mitomycin C were commonly used chemotherapy agents emulsified in the lipiodol solution [1,22]. However, doxorubicin alone is more frequently used due to shortages of the other agents. Lipiodol acts as an embolic material, helps increase drug concentration at the tumor site, and acts as a contrast agent allowing for visualization during intra-arterial delivery [1,9]. DEB-TACE involves the intra-arterial administration of embolic microspheres coated with chemotherapy. The administration of these coated microspheres allows for the simultaneous embolization of a tumor’s blood supply and direct delivery of chemotherapy. DEB-TACE is proposed to increase drug concentration at the tumor and avoid systemic leakage of chemotherapy, combating described limitations and adverse effects of cTACE [25,26]. Multiple microspheres for DEB-TACE exist, with various size ranges and biochemical properties. As demonstrated by DC-Bead (BTG International, London, UK) which are hydrogel microspheres ranging in sizes from 70 to 150, 100–300, 300–500 and 500–700 um and HepaSphere (Merit Medical, South Jordan, UT, USA) which are expandable microspheres ranging from 30 to 60, 50–100, 100–150 and 150–200 um in the dry state [27], safe and effective results have been demonstrated across studies with various agents and sizes [28].

The selection of cTACE or DEB-TACE remains controversial, with numerous studies attempting to elucidate improved outcomes or decreased adverse effects with one technique over the other. Early RCTs found no significant differences between the two therapies in intermediate-stage HCC (Table 1). However, more recently, improved outcomes with DEB-TACE in cases of HCC with portal vein tumor thrombus have been reported (Table 1). At the meta-analysis level there are also conflicting results, with some studies showing no significant difference between the two therapies, while others have shown improved outcomes with DEB-TACE. In a recent meta-analysis, improved OS rates and relapse-free survival rates (RFS) were found with DEB-TACE over cTACE in unresectable HCC (Table 1). In contrast, a separate meta-analysis for unresectable HCC found OS rates were not statistically different between DEB-TACE and cTACE; however, objective tumor response rates were higher with DEB-TACE (Table 1).

**Figure 1:**
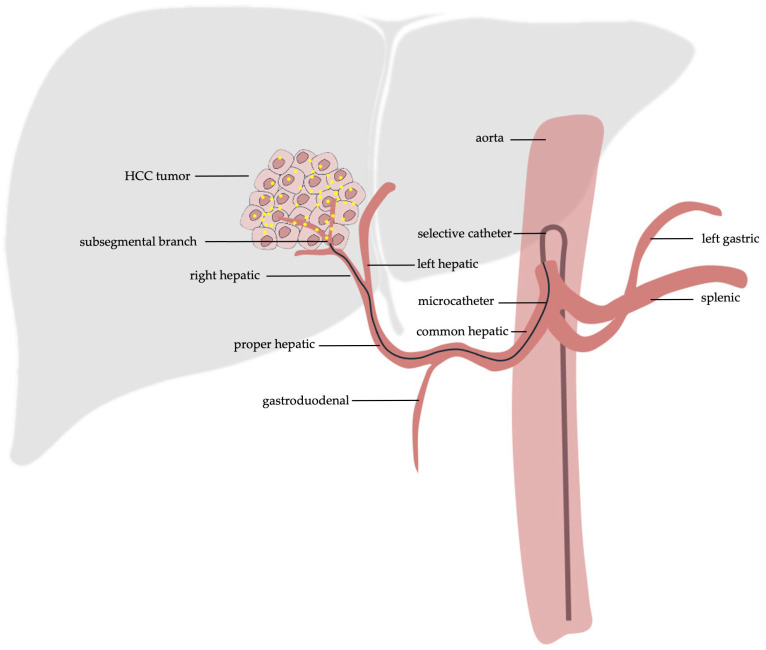
Original illustration of the general technique used in TACE. In the absence of variant anatomy, the celiac artery is first selected. A microcatheter is then inserted to select the tumors arterial supply for administration of the therapy. Lobar, selective or superselective administration can be performed, with the illustration demonstrating superselective technique. Yellow dots within the tumor depicting the agent for chemoembolization.

The procedural steps are largely similar in cTACE and DEB-TACE. Arterial access is obtained first, followed by selection of the celiac artery with a selective angiographic catheter, assuming no variant anatomy. After confirming appropriate selection, a microcatheter is commonly used to select the tumor’s arterial supply, which can be further evaluated with cone beam computed tomography. Then, the lipiodol and chemotherapy agent combination are infused in cTACE or the coated drug-eluting beads in DEB-TACE (Figure 1). Fluoroscopy is used during injection to check for adequate stasis of flow and monitor for non-target embolization. Optionally, a balloon-occlusion catheter can be used to help reduce leakage of agents and increase concentration of the delivered therapy at the desired site in either cTACE or DEB-TACE. Following successful chemoembolization, the arterial site is closed, most frequently with an arterial closure device or alternatively with manual pressure. Depending on patient factors and institutional preference, patients may be discharged the same day or kept for overnight admission. Typically, patients will have a clinical follow-up and repeat imaging in 8–12 weeks after treatment [9].

### 2.2. Superselective TACE

The TACE technique can be modified according to the site of arterial therapy administration as either lobar, selective, or superselective. Lobar TACE is commonly considered as an injection in a proximal hepatic artery to broadly target the left or right hepatic lobe depending on the tumor(s’) location [34]. As such, increased doses of chemotherapy and embolic agents must be used to achieve therapeutic effect. However, these increased doses can be hepatotoxic and damage healthy hepatic tissue as they are released into the general hepatic circulation [35]. By dispersing the pharmacological agents further from the tumor bed, there is an increased risk of embolizing additional arteries that do not directly feed the tumor, potentially damaging blood supply to healthy hepatic tissue [35].

To circumvent these side effects, selective and superselective TACE procedures have been developed, wherein catheters are advanced past the proximal hepatic arteries and directed closer to the tumor bed. Specifically, these procedures are defined by the depth at which the catheters are inserted into the hepatic vasculature. Selective TACE is often defined as the application of TACE at a segmental hepatic artery. Superselective TACE is defined as the application of TACE directly at the subsegmental hepatic arteries [1]. This allows for more targeted delivery of chemotherapy and embolic agents to the tumor site, allowing for lower doses to be used with greater efficacy [35]. Selectively targeting the specific arteries that feed the tumor results in less involvement of healthy hepatic tissue, reducing overall hepatic damage while maintaining adequate tumor necrosis in a majority of cases [36]. Studies have validated the effectiveness of superselective TACE with significant increases in survival rates, preservation of liver function and decreases in tumor sizes and numbers being reported [37]. Multiple studies advocate that superselective TACE should be the first option considered when discussing a potential TACE procedure for a patient [38,39,40]. Common considerations include tumor size, number of tumors, and location of the tumor(s) in the decision to recommend superselective TACE. Exact criteria for this recommendation vary between reports, with some recommending superselective TACE for tumors with diameters less than 7 cm, while others use criteria such as fewer than 5 lesions across and fewer than 4 separate hepatic segments [41,42].

### 2.3. Balloon-Occluded TACE

B-TACE is an adjunct technical option in the procedure of TACE using a balloon-occlusion catheter to deliver the therapeutic agent. These catheters, which cause occlusion through the inflation of a balloon at the distal end, can be used in both cTACE and DEB-TACE. The benefits with occlusion of the selected tumor’s arterial supply during the injection of therapy are thought to be secondary to the alteration of hemodynamic properties [43]. In B-TACE, reducing the balloon-occluded arterial stump pressure allows for the powerful injection of therapeutic agents, allowing for improved penetration. Occlusion of the artery also prevents backflow of chemotherapy agents, reducing leakage outside of the target by creating stagnant arterial blood flow [43]. The stagnant environment created in B-TACE, in contrast to the free-flowing infusion with non-balloon cTACE or DEB-TACE, reduces systemic delivery of the chemotherapy agents, while enhancing drug concentration at the tumor site [43].

Multiple studies have demonstrated improved effectiveness of B-TACE over non-balloon-occluded techniques. As demonstrated in a comparison of B-TACE to non-balloon occluded cTACE for the delivery of miriplatin in HCC, B-TACE demonstrated improved outcomes [44]. Specifically, patients in the B-TACE group experienced a higher average treatment effect of 55.1% compared to the 39.6% seen in the matched patients treated with non-balloon occluded cTACE. Specifically, improved miriplatin delivery to the target site was demonstrated [44]. When comparing B-TACE against non-occluded TACE for HCC tumors of various sizes, B-TACE performed better. In a multicenter study, regardless of HCC size, an increased initial complete response rate of 59.3% in a B-TACE group compared to 49.5% in a non-occluded TACE group was demonstrated [45]. The improved efficacy of B-TACE was even more pronounced in intermediate-sized tumors (30–50 mm), where the B-TACE group saw an average initial complete response rate of 72.3% compared to 54.1% in the non-occluded TACE group [45].

Adverse outcomes of B-TACE have been reported at a similar rate to counterpart non-occluded TACE techniques [46,47]. While improved results and similar safety profiles are promising for many clinical scenarios, it is notable that B-TACE has not been shown to increase concentration of emulsion in cases of innumerable HCC tumors [48].

## 3. TACE and Combination Therapies

### 3.1. TACE and Radiation Therapy

TACE combined with radiation therapy (TACE-RT) aims to take advantage of both therapies to create a synergistic effect in hopes of improving survival outcomes with HCC treatment. TACE is typically performed first to create a hypoxic and ischemic environment, which enhances the subsequent effects of radiation therapy. Chemoembolization not only disrupts the tumor’s vascular supply, but also sensitizes tumor cells to radiation by disrupting repair mechanisms and inducing cellular stress [49]. Subsequent radiation therapy after TACE increases DNA damage of tumor cells, ultimately inhibiting their ability to grow and divide [49]. In this fashion, TACE-RT is proposed to offer improved outcomes for HCC patients compared to either intervention alone. Multiple studies have demonstrated proven benefit with combination TACE-RT compared to monotherapy. Dumago et al., in a recent systematic review and meta-analysis, reported improved outcomes in patients receiving TACE-SBRT versus TACE alone [50]. Specifically, improved 1- and 3-year PFS with combination therapy compared to TACE alone (56.5% and 32.3% compared to 42.2% and 21.6%) [50]. Statistical pooling of the included retrospective studies also demonstrated improved 3-year OS in the TACE-SBRT group [50]. Huo et al., in a systematic review and meta-analysis involving 25 trials with 2577 total patients, found TACE-RT had an improved 1-year survival rate with a progressively increasing survival benefit at 2, 3, 4 and 5 years [51]. Chen et al., in a recent randomized study, included patients with unresectable HCC for comparison of TACE therapy alone against TACE and SBRT [52]. Improved outcomes were found with the combination therapy group, having a 75% complete response rate compared to 54.5% in the TACE monotherapy group [52]. Furthermore, the combination therapy group had longer median PFS of 16 months compared to 11 months in the TACE monotherapy group [52]. TACE-RT has also demonstrated potential improved therapeutic benefits in patients with more advanced disease who traditionally have more limited treatment options. HCC patients with macroscopic vascular invasion are among this group for which systemic treatment with sorafenib was previously the mainstay of treatment [53,54]. In a randomized open-label clinical trial comparing the efficacy of TACE-RT against treatment with sorafenib for HCC patients with macroscopic vascular invasion of the portal vein, significantly improved response was demonstrated in the combination therapy group [54]. Specifically, TACE-RT demonstrated a higher progression-free survival (PFS) rate at 12 weeks of 86.7% compared to 34.3% in the sorafenib group [54]. TACE-RT patients also had a significantly longer OS at 55 weeks compared to 43 weeks in the sorafenib group, and curative surgical resection was performed in 11.1% of the TACE-RT group compared to 0% in the sorafenib group [54].

Radiation therapy following TACE typically involves stereotactic body radiotherapy (SBRT), a form of external beam radiation therapy (EBRT). In contrast to conventional EBRT which involves a dose rate of 1.8–2 Gy per fraction delivered over weeks, SBRT delivers high doses of radiation with sub-millimeter precision in fewer fractions. The intention of SBRT is to treat with higher doses in fewer sessions in a rapid fall-off technique to reduce damage to surrounding structures [55]. There is some heterogeneity in the radiation protocols described in combination TACE-RT therapy. However, general dose and fractionation ranges are similar across reports and recommendations. Typically, 25–50 Gy is delivered in 3–10 fractions over 1–2 weeks [55]. This is concordant with the national comprehensive cancer network guidelines for HCC and liver metastases which recommend 27.5–50 Gy in 3–5 fractions [56].

In combined TACE-RT therapy, timing of RT following TACE varies across studies. However, the first RT treatment is commonly 1 month or less following TACE. In a recent prospective study, patients received SBRT 1 month following TACE treatment with good clinical outcomes [56]. Separately, a RCT comparing TACE-RT against TACE alone implemented a shorter interval of RT treatment beginning 10–15 days after TACE with good results [57]. Irrespective of the RT treatment interval following TACE, results across studies are generally concordant with improved clinical outcomes with combination therapy when compared to TACE monotherapy. A large systematic review and meta-analysis specifically analyzed the time interval across studies by comparing a TACE-RT interval of more than 28 days to less than 28 days [51]. One-year survival rates, response rates and complete response rates were not statistically different between patients who received RT 28 days or more after TACE compared to 28 days or less [51]. Regardless of the interval, TACE-RT was more effective than TACE alone, specifically with respect to 1-year survival and complete response rates [51].

Radiation-induced liver disease (RILD) is the major potential adverse effect associated with SBRT therapy. Generally, RILD is divided into two forms, referred to as classic RILD and non-classic RILD. Classic RILD involves anicteric hepatitis, ascites, and elevated liver enzymes. Non-classic RILD manifests as elevated serum transaminase levels and a worse Child-Pugh score of two or greater [55]. Generally, major adverse effects are not reported to be increased in combination TACE-RT groups. In the randomized study by Chen et al., a few patients in the group receiving SBRT demonstrated liver function deterioration at one month; however, this was temporary, with most recovering by three months [52]. A total of 10% of patients in the combination therapy group deteriorated from Child-Pugh A to B compared to 4.54% in the TACE monotherapy group, which was not statistically significant. Two patients in the combination therapy developed grade 2 RILD. The study concluded that transient acute liver toxicity may be found with SBRT combination therapy; however, this is often reversible [52]. Huo et al. in the systematic review and meta-analysis reported the adverse events of gastroduodenal ulcers; increased alanine aminotransferase levels, and increased total bilirubin levels were found in TACE-RT combination therapy compared to TACE alone [51]. Additional outcome measurements mentioned in the trials included decline of leukocyte count, nausea and/or vomiting, thrombocytopenia, fever, and esophagitis or gastroduodenitis. However, TACE plus radiotherapy in the pooled analyses did not significantly increase these adverse events compared to TACE alone [51].

### 3.2. TACE and Ablation

Ablation therapies for HCC are a group of locoregional target-directed therapies. Local ablative therapies for HCC include radiofrequency ablation (RFA), microwave ablation (MWA), cryoablation and chemical ablation [9,58]. Typically, they are indicated and reserved for small tumor sizes and as a bridging therapy to transplantation [59]. In the early stage of disease, ablative therapies have shown similar survival rates to surgical resection, making ablation an option for those who are poor surgical candidates [59].

In RFA, electromagnetic radiation generates thermal energy at the target site through percutaneous probes [9]. RFA is currently indicated for HCC in BCLC early-stage, and when combined with other locoregional therapies for BCLC intermediate-stage [9]. Variable sizes and numbers of probes can be used to fine-tune treatment; however, typical use is for tumors up to 3 cm in size, with some evidence for tumors up to 5 cm in size [9]. In MWA, microwaves are used to cause thermal damage, similar to RFA, with percutaneous probes. The advantage of MWA over RFA is the ability to better control the zone of ablation [9]. In cryoablation, rapidly cooling tumor tissue with freeze–thaw cycles results in tissue death. Similarly to RFA and MWA, percutaneous probes are used in cryoablation, but the addition of multiple probes allows a synergistic effect to treat lesions up to 9 cm in size [9]. In chemical ablation, tissue damage is typically achieved with local delivery of ethanol or acetic acid. Lesions of 1–3 cm can be treated, but multiple therapy sessions are required with chemical ablation [9].

Combination therapy with ablation and TACE has shown promising results by overcoming limitations of each respective treatment. TACE and ablation aim to increase the size of tumors that can be treated to expand treatment options and improve outcomes [59]. A considerable limitation of RFA is the phenomenon of blood flow dissipating the thermal energy delivered to the tumor, thus reducing theoretical effectiveness. With RFA combined with TACE, a principal benefit is thought to result from the reduced blood flow induced by TACE, resulting in this decreased heat sink effect [59]. This has allowed for expansion and increased effectiveness of the RFA treatment ablation zone. As demonstrated in a head-to-head comparison of 189 patients with HCC who had a single tumor up to 7 cm or in the case of multiple tumors (up to 3 with each up to 3 cm), there was statistically significant improvement in 4-year survival with TACE combined with RFA versus RFA alone [59]. Multiple meta-analyses have shown similar results with better OS rate with combination therapy versus either therapy alone [59]. While thermal ablative therapies have the best prognostic outcomes for tumors up to 3 cm in size, it has been postulated that with the combination of TACE, lesions at this border zone size or larger would have a better response. A recent retrospective review evaluating the combination of thermal ablation with TACE for either bridging therapy to liver transplant or downstaging purposes prior to transplant showed increased effectiveness of combination treatment for average tumor sizes of 4.25 cm [60]. Similarly, a large meta-analysis including 1892 patients comparing TACE combined with RFA against surgical resection found that 1-, 3-, and 5-year OS rates were similar between groups [61]. Likewise, when comparing the combination of TACE with MWA against TACE with cryoablation, similar efficacy existed between the two groups in the setting of unresectable HCC [62]. However, when TACE was combined with MWA, there were fewer complications as compared to TACE combined with cryoablation [62].

### 3.3. TACE with Targeted Therapies and Immunotherapies

The frequently late-stage presentation of HCC often results in limited treatment options, where systemic options of targeted therapies and immunotherapies have been a mainstay in management. Success with systemic therapies ultimately led to investigations on combination therapy with TACE, aiming to improve therapeutic response.

Current targeted therapies and immunotherapies for HCC primarily target the vascular endothelial growth factor (VEGF), the tyrosine-kinase pathway, programmed cell death protein 1 (PD-1), programmed cell death ligand 1 (PD-L1), and cytotoxic T-lymphocyte-associated protein 4 (CTLA-4) [63]. The tyrosine-kinase inhibitor sorafenib was previously the primary recommended systemic therapy for advanced HCC owing to success in the SHARP trial (Table 2). Further supporting tyrosine-kinase inhibitors, lenvatinib in the REFLECT trial was shown to be non-inferior to sorafenib (Table 2). Involving PD-L1 and the VEGF, the PD-L1 checkpoint inhibitor atezolizumab combined with the VEGF inhibitor bevacizumab demonstrated improved OS and PFS compared to sorafenib in the IMbrave150 trial (Table 2). Regarding CTLA-4, the anti-CTLA-4 agent tremelimumab combined with the anti-PD-L1 agent durvalumab demonstrated improved OS compared to sorafenib in the HIMALAYA trial (Table 2).

Combining targeted therapies and immunotherapies with TACE aims to address changes induced following TACE therapy and take advantage of previously relatively protected tumor-associated antigens. As a consequence of exacerbating tumor hypoxia after TACE, pro-angiogenesis mechanisms are upregulated following chemoembolization, where VEGF and tyrosine-kinase inhibitors can be used to combat this upregulation. In addition to increased tumoral antigen exposure, TACE has been shown to decrease tumoral exhausted immune cells and increase inflammatory pathways [68]. In this fashion, part of TACE efficacy owes to its effects on the innate and adaptive immunity [69]. This has been demonstrated with phenotypic lymphocyte characterization in liver samples of patients who underwent TACE where intratumoral exhausted effector cytotoxic and regulatory T-cells were found in lower concentration and pro-inflammatory pathways were upregulated [69]. Specifically, lower CD4+/FOXP3+, CD8+ and CD8+/PD-1+, and NT CD8+/PD-1+ were found in the samples from the TACE-treated patients compared to samples without TACE therapy [69]. With this known effect, the combination of TACE with immunotherapy has been a focus of recent and ongoing studies. The EMERALD-1 trial investigated TACE combination therapy by comparing TACE plus durvalumab or durvalumab with bevacizumab against TACE alone in patients with unresectable HCC. In EMERALD-1, PFS was significantly increased in the durvalumab with bevacizumab plus TACE combination group compared to TACE alone (Table 3). The LEAP-012 trial compared TACE plus lenvatinib with pembrolizumab against TACE alone in patients with unresectable HCC. Improved median PFS was demonstrated in the TACE combination therapy group compared to the TACE with placebo group. While longer follow-up data is needed, OS was also increased in the TACE combination therapy group (Table 3). In a similar fashion to these described pharmaceuticals, the same concept has been demonstrated with traditional Chinese medicine utilizing the Jiedu Recipe [70]. Tumor-derived exosomes are known to be involved in tumor processes such as angiogenesis and metastasis, and are upregulated in response to hypoxia such as what is seen post-TACE [70]. A bioinformatics analysis and in vitro assay have demonstrated the ability of the Jiedu Recipe to inhibit hypoxia-induced exosome release, which may be beneficial in the post-TACE setting [70].

Novel immunotherapies will be continually developed, and combination treatments will continue to undergo high-level investigations to validate this work. Looking ahead, EMERALD-3, a phase III randomized multicenter study, is investigating durvalumab plus tremelimumab with or without lenvatinib in combination with TACE compared to TACE alone in HCC without extrahepatic metastatic disease (Table 3). With an expected completion date in early 2027, the three comparative arms will provide further information on combination therapy.

## 4. TACE Prognostic Models

### 4.1. Radiomics

In addition to TACE procedural advancements, increased attention is being directed towards the development of machine learning models for predictive and prognostic assessments on HCC treatment. In HCC, predictive models attempt to understand which patients would most likely benefit from TACE, while prognostic models are aimed at determining patient response after TACE. Prognostic models have the potential to identify which patients may require repeat or even alternate intervention [74]. Radiomics is an evolving field that attempts to provide this information by extracting data from imaging that which is typically considered unquantifiable by the human eye. Properties such as shape, heterogeneity, intensity, distribution, and texture are among some of the information different radiomic models use. Tumor heterogeneity, for example, when analyzed at the cellular and genomic level, is known to hold valuable prognostic implications. Various radiomics models have been capable of identifying tumor heterogeneity that strongly correlates with actual heterogeneity at the cellular level [75]. Radiomics can not only capture this information in a non-invasive fashion, but can also obtain this data throughout the entire lesion, as opposed to a traditional biopsy which is both invasive and provides only limited tissue samples. While heterogeneity provides proven beneficial prognostic information, the mineable property of data with radiomic systems allows for potentially novel patterns to be uncovered. These patterns, previously unrecognized or undetectable, could provide more powerful predictive or prognostic information [75,76].

There are various radiomic systems in development and undergoing validation for the ability to determine HCC response after TACE. Multiple reviews have analyzed these machine learning models for HCC treatment and found they have performed well at accurately predicting response after TACE [77,78]. In a recent systematic review and meta-analysis, 24 different studies were analyzed for their capability of determining therapeutic response and survival status for patients with HCC after TACE. Models analyzed included what are referred to as radiomics-clinical models, radiomics models, and clinical models, implementing the respective parameters. The radiomics-clinical model was found to perform the best, with concordance indexes of 0.93 and 0.88 for therapeutic response and 0.84 and 0.80 for survival status in training and validation sets, respectively [74]. Another representative radiomic prognostic tool described is the use of radiomics in the prediction of HCC extrahepatic metastasis after TACE intervention. Utilizing pre-operative magnetic resonance imaging (MRI) for patients undergoing TACE, radiomics machine learning models have shown effectiveness in predicting extrahepatic metastasis [76]. Specifically, radiomics machine learning models and the combination of these models with clinical parameters have proven better performance than clinical models alone [76]. While these models only serve to represent portions of the population, more work is needed to develop current machine learning models to accurately represent the entire HCC patient population [76]. Additionally, many of these predictive models will need external validation before larger and widespread use [77]. A part of future work will also work on combating the commonly discussed limitations of these models, including image acquisition and class imbalances. As with any system relying on image analysis, high-quality images obtained under similar protocols and reconstruction methods are required for many of these radiomics models. Either uniformity in these parameters or more likely the ability of the radiomics models to adapt to differences in image acquisition and reconstruction will be required. Class imbalances, where the frequency of a finding is far less than the absence of that finding, can result in models being falsely labeled as incapable of detecting that finding. Models and the reported research on their performance will need to account for these imbalances and report the appropriate statistical analyses validating these results [75].

### 4.2. Neutrophil-to-Lymphocyte Ratio (NLR)

Cellular inflammatory markers have gained attention recently for prognostic evaluation in cancer treatment, in part due to an increased understanding of their roles in cancer pathogenesis. Various scores and ratios have been developed, including the neutrophil-to-lymphocyte ratio (NLR), lymphocyte-to-monocyte ratio and the lymphocyte-to-C-reactive protein ratio. The NLR has gained particular interest given the strong association of neutrophils with cancer pathogenesis and progression. Neutrophils are known to promote inflammatory pathways that are thought to contribute to cancer pathogenesis, cause DNA damage, promote angiogenesis and cause immunosuppression. In relation to carcinogenesis, the pro-inflammatory neutrophil extracellular traps (NETs) are known to promote HCC development in at-risk patients. NETs contribute to naïve CD4+ T-cell differentiation from regulatory T-cells, which has been related to HCC formation. Additionally, neutrophils are known to aid in metastatic disease with their role in cancer cell migration. In contrast, lymphocytopenia can portend a poor prognosis, partially owing to weak cell-mediate immunity. The NLR is thus proposed to provide valuable prognostic implications in HCC [79].

Several studies have been conducted to evaluate the efficacy of the NLR as a prognostic model for HCC. A retrospective examination of 380 patients with HCC treated with TACE found that lower NLR values correlated with improved OS, whereas higher NLR values were associated with worse OS [80]. These findings were further corroborated by a study aimed at developing and validating a prognostic model from 931 patients with HCC. NLR data from 495 patients assigned to the development group was used to develop a prognostic model, found capable of accurately assessing HCC prognoses of the 436 patients in the validation group [81]. In this fashion, the NLR could be used alone or in combination with other prognostic assessments to help guide patient-management decisions. While early reports on the NLR have been promising, subsequent studies will need to thoroughly account for several inherent limitations. Neutrophils and lymphocytes are affected by a variety of factors, including infections, inflammatory states and medications [79,80], all of which vary between patients and often continuously change for individual patients. Controlling for these differences and attributing changes in the neutrophil and lymphocyte values as they relate to the HCC disease process despite these background influences will need to be considered in future studies.

### 4.3. Albumin-Bilirubin Grade

A HCC prognosis is known to be influenced by underlying liver function, as is emphasized with the inclusion of liver function status in staging systems with the widely used Child-Pugh score. The albumin-bilirubin (ALBI) grade is a relatively new model for assessing liver function using only albumin and bilirubin to categorize patients with HCC into one of three prognostic groups [82]. With HCC prognosis affected by liver function, ALBI grading is proposed to provide an accurate and reliable prognostic system by relying on objective measures only. In contrast, the commonly used Child-Pugh score utilizes the subjective components of ascites and encephalopathy [82]. In patients with HCC, a recent systematic review and meta-analysis found a statistically significant relationship between an elevated ALBI grade and decreased OS. In this study, Xu et al. reported a significant HR of 1.71 for grade 1 vs. grade 2 and a HR of 3.81 for grade 1 vs. grade 3 [82]. The ALBI grade was also shown to distinguish patients with Child-Pugh score A into two separate ALBI grades of grade 1 and grade 2, with associated individual survival outcomes. This validated the prognostic implications of the ALBI grading system and also highlighted its potential to provide more distinct prognostic information for patients [82]. Furthermore, ALBI score trajectories have shown to be independent risk factors for OS in patients with intermediate and advanced stage HCC post-TACE intervention [83]. Despite the support for utlizing solely objective factors in the grading system, concern could arise from the limited factors used with only two biomakers utilized to depict liver function. The potential for other physiologic factors that may influence these biomarker levels will need to be considered. Future work could involve the integration of the ALBI grade into staging systems for potential improved predictive and prognostic information, which should be directly compared to existing models.

### 4.4. Assessment for Retreatment with TACE Score

The Assessment for Retreatment with TACE (ART) score is a prognostic model used to help guide the decision for retreatment after TACE in HCC. The Child-Pugh score, AST, and radiologic assessment of tumor response are used to calculate a score to determine if a subsequent TACE treatment would be safe and beneficial for the patient. A score is assigned to each of these parameters, and based on the sum, the system suggests that either TACE may be beneficial or that TACE should be avoided and consideration for alternate therapy recommended. Multiple separate studies have validated this scoring system, showing that patients who had ART scores of less than 1.5 as compared to ART scores greater than 2.5 had better outcomes with repeat TACE interventions [84,85]. Variations in the ART score have been proposed in an attempt to improve the prognostic assessment. It has been suggested that incorporating thrombocytopenia into the score would be beneficial owing to the fact that thrombocytopenia is commonly encountered with liver cirrhosis. A prognostic system referred to as P-ART integrates platelet count into the ART score by assigning additional points for a decrease in platelets of at least 10,000 and creating a new cutoff value of less than 5 or greater than 5.5. The P-ART system has demonstrated the ability to accurately predict significant differences in survival for TACE re-intervention [86]. Common to similar models, factors that may affect platelet counts outside of HCC, such as comorbid conditions and medications, will need to be accounted for during score assessment. The clinical utility of the modified ART system with P-ART or alike models will require further validation with larger data before adoption into standard HCC treamtent decisions.

## 5. Future Directions

Further advances and validation with TACE combination therapy will continually change the treatment landscape for HCC, as will the ability to select patients for intervention and predict response with future work in the field of prognostic applications. In addition to these new therapies and models, work is continuously being done to advance the technical aspects of the TACE procedure itself. While TACE benefits are relatively well supported, the catheter-directed nature of the procedure is partially limited by the altered tumor microenvironment. Device advancement for catheter-directed delivery of therapeutics is underway to improve efficacy and limit adverse effects of these interventions. One such device advancement is the development of catheters capable of pressure-enabled drug delivery. High intra-tumoral pressure limits the ability of therapeutics to penetrate deep within a tumor, a limitation to traditional microcatheter delivery. It is proposed that modulating pressure and flow within the target vessel through the use of catheters capable of pressure-enabled drug delivery allows for more uniform therapeutic administration with increased concentrations in the targeted tumor [87]. One such device is the TriNav catheter (TriSalus Life Sciences; Westminster, CO, USA), which uses a novel valve to create pressure-enabled therapeutic delivery of material. The use of this catheter is proposed to self-center for improved particle distribution, create turbulent flow for greater particle mixing, and allow for pressure modulation to combat high intra-tumoral pressure for improved penetration. Early investigations have reported promising results. A 100% objective response rate and 88.8% pathological response rate with the TriNav pressure-enabled catheter was reported in comparison to 76.5% and 33.8%, respectively, with traditional microcatheters [88]. However, while the reported results are encouraging, it is important to note they are derived from a single-center retrospective study with a small patient sample, inherently limiting the power and generalizability of the results. Early research has also commonly reported increased penetration of microspheres with pressure-enabled drug delivery catheters compared to conventional catheters. This data is derived from a porcine-induced liver tumor model and further validation will be required in the HCC setting [89]. To date, no prospective RCT has been conducted investigating the technology. In order to recommend widespread adoption of pressure-enabled drug delivery devices, the efficacy and safety of the technology will need to be confirmed with higher-level evidence.

Advances in catheters can be complemented with the research being done on new embolic agents. In contrast to permanent embolic agents, a category of degradable temporary embolic material referred to as biodegradable embolic agents is being developed. Degrading at various rates, temporary embolic materials are proposed to increase options for re-treatment intervention and importantly decrease liver damage. Additionally, these embolic agents have the capacity for drug loading with various chemotherapies, and recent research is investigating the dual incorporation of loading these embolic materials with multiple different agents. In this way, embolic materials could contain anti-VEGF agents to combat the upregulated pro-angiogenic mechanisms in the post-TACE environment, in addition to their chemotherapy effects [90].

TACE therapy will be further augmented by accurate predictive and prognostic models. In addition to machine learning models and the field of radiomics, the development of biomarker signatures is under investigation with the aim of predicting HCC response to TACE. It is proposed that studying biomarkers may be more accessible and less resource-intensive than different models such as the radiomics-based methods used to predict response to TACE. A recent study performed a bioinformatics analysis utilizing a gene expression database from tumor samples of TACE responders and non-responders to try and identify a reliable biomarker signature. Ultimately, six genes (CXCL8, AFP, CYP1A1, MMP9, CYP3A4 and SERPINC1) were identified to have predictive value in HCC response to TACE [91]. Another such example is with the analysis of micro-RNAs (miRNAs). The miRNA biomarkers are small, non-coding RNA molecules that serve vital cellular functions, and their dysregulation is linked to cancer pathogenesis [92]. A recent meta-analysis studied numerous miRNA molecules and demonstrated a pooled sensitivity in predicting recurrence after TACE of 0.79 with a specificity of 0.82. Notably, in this meta-analysis, some miRNAs thought to function as oncogenes displayed decreased expression after TACE, such as miR-210 and miR-373. Further work will focus on further delineating the relationship of these miRNAs and other alike biomarkers [92].

## 6. Conclusions

HCC continues to be one of the leading causes of cancer-related death, with a poor long-term survival rate and often late-stage diagnosis at presentation. The locoregional therapy TACE is a focus of ongoing and future research, as it offers treatment for patients who previously had limited options due to the aggressive nature of HCC. Early advances came from technique adjustments with the advent of DEB-TACE, and more selective therapy administration beyond the traditional lobar approach. Now, with promising investigations on TACE combined with radiation, ablation, and immunotherapy, treatment options continue to expand. Future work will further investigate and validate these combination treatments along with novel prognostic models and procedural techniques.

## Figures and Tables

**Table 1:** Data comparing efficacy of cTACE vs. DEB-TACE in HCC treatment.

Author, Year	Study Design	Outcomes		
		Measure	95% CI	*p*
Golfieri et al., 2014 [29]	RCT DEB-TACE vs. cTACE in intermediate HCC, 177 patients	Local tumor response: no difference	-	0.05
		Overall tumor response: no difference	-	0.05
		Median TTP cTACE: 9 months	(6.3, 11.7)	0.766
		Median TTP DEB-TACE: 9 months	(6.8, 11.2)	0.766
Chen et al., 2017 [30]	Meta-analysis, DEB-TACE vs. cTACE in unresectable HCC, 16 studies including 1832 patients	1-year OS RR: 1.12	(1.03, 1.23)	0.007
		2-year OS RR: 1.26	(1.03, 1.54)	0.02
		3-year OS RR: 1.69	(1.00, 2.84)	0.04
		1-year RFS: 1.21	(1.01, 1.44)	0.03
		2-year RFS: 1.68	(1.17, 2.43)	0.005
		3-year RFS: not statistically significant	-	-
Wang et al., 2023 [31]	Meta-analysis, DEB-TACE vs. cTACE in unresectable HCC, 24 studies including 2987 patients	1-year OS RR: 1.05	(0.99, 1.11)	0.80
		2-year OS RR: 1.02	(0.93, 1.11)	0.68
		3-year OS RR: 0.92	(0.77, 1.10)	0.37
		5-year OS RR: 0.92	(0.47, 1.80)	0.81
		Objective tumor response rate RR: 1.27	(1.08, 1.48)	0.003
Zhou et al., 2024 [32]	RCT DEB-TACE vs. cTACE in HCC with portal vein tumor thrombus, 163 patients	Median PFS HR: 0.63	(0.42, 0.95)	0.027
		Median OS DEB-TACE: 12 months	(9.0, 16.0)	0.039
		Median OS cTACE: 8 months	(7.0, 11.0)	0.039
Chernyshenko et al., 2025 [33]	Meta-analysis, DEB-TACE vs. cTACE in HCC, 32 studies including 4367 patients	OS DEB-TACE vs. cTACE: 3.54 months	(2.10, 4.98)	0.00001
		PFS DEB-TACE vs. cTACE: 3.07 months	(1.66, 4.49)	0.0001

CI, confidence interval; HR, hazard ratio.

**Table 2:** Landmark trials for targeted therapies and immunotherapies in HCC treatment.

Trial, Year	Design	Trial Arms	Outcomes
SHARP, 2008 [64]	Multicenter, phase 3, double-blind, placebo-controlled trial with unresectable advanced HCC	sorafenib vs. placebo	sorafenib improved median OS (10.7 months vs. 7.9 months) [HR: 0.69; 95% CI: 0.55–0.87; *p* < 0.001]
			sorafenib improved median time to radiological progression (5.5 vs. 2.8 months; *p* < 0.001)
REFLECT, 2018 [65]	Multicenter, phase 3, open-label, non-inferiority trial with unresectable HCC	lenvatinib vs. sorafenib	Median survival time of 13.6 months for Lenvatinib (95% CI 12.1–14.9) and 12.3 months for sorafenib (95% CI 10.4–13.9), (HR 0.92, 95% CI 0.79–1.06), demonstrating non-inferiority
IMbrave150, 2020 [66]	Global, phase 3, open-label trial with unresectable HCC.	atezolizumab plus bevacizumab vs. sorafenib	12-month OS: 67.2% (95% CI, 61.3 to 73.1) with atezolizumab–bevacizumab
			12-month OS: 54.6% (95% CI, 45.2 to 64.0) with sorafenib
			Median PFS: 6.8 months (95% CI, 5.7 to 8.3) with atezolizumab–bevacizumab
			Median PFS: 4.3 months (95% CI, 4.0 to 5.6) with sorafenib
			(HR for disease progression or death, 0.59; 95% CI, 0.47 to 0.76; *p* < 0.001)
HIMALAYA, 2022 [67]	Global, phase 3, open-label trial with unresectable HCC	tremelimumab plus durvalumab (STRIDE) vs. durvalumab vs. sorafenib	Median OS: 16.43 months (95% CI, 14.16–19.58) with STRIDE
			Median OS: 16.56 months (95% CI, 14.06–19.12) with durvalumab
			Median OS: 13.77 months (95% CI, 12.25–16.13) with sorafenib
			OS at 36 months: 30.7% with STRIDE
			OS at 36 months: 24.7% with durvalumab
			OS at 36 months: 20.2% with sorafenib
			OS HR for STRIDE vs. sorafenib was 0.78 (96.02% CI, 0.65–0.93; *p* = 0.0035)
			OS HR for durvalumab vs. sorafenib 0.86 (95.67% CI, 0.73–1.03)

**Table 3:** Major trials investigating TACE in combination with targeted therapies and immunotherapies for HCC treatment.

Trial, Year	Design	Trial Arms	Outcomes
EMERALD 1, 2025 [71]	Multiregional, phase III, randomized, double-blinded and placebo-controlled trial for unresectable HCC	TACE plus durvalumab with bevacizumab vs. TACE plus durvalumab vs. TACE	median PFS: 15.0 months (95% CI 11.1–18.9) with TACE plus durvalumab with bevacizumab
			median PFS: 10.0 months (9.0–12.7) with TACE plus durvalumab median PFS: 8.2 months (6.9–11.1) with TACE
			PFS HR was 0.77 (95% CI 0.61–0.98; two-sided *p* = 0·032) for TACE plus durvalumab with bevacizumab vs. TACE, and 0.94 (0.75–1.19; two-sided *p* = 0·64) for TACE plus durvalumab vs. TACE
LEAP-012, 2025 [72]	Multicenter, phase III, randomized and placebo controlled double blinded trial for unresectable HCC	TACE plus lenvatinib and pembrolizumab vs. TACE	Median PFS: 14.6 months (95% CI 12.6–16.7) with TACE plus lenvatinib and pembrolizumab Median PFS: 10.0 months (95% CI 8.1–12.2) with TACE HR 0.66 (95% CI 0.51–0.84); one-sided *p* = 0·0002
			24-month OS: 75% (95% CI 68–80) with TACE plus lenvatinib and pembrolizumab 24-month OS: 69% (62–74) with TACE HR 0.80 (95% CI 0.57–1.11); one-sided *p* = 0·087
EMERALD 3, expected 2027 [73]	Multicenter, phase III randomized, open-label trial for HCC without extrahepatic metastatic disease.	TACE plus tremelimumab with durvalumab (STRIDE) with or without lenvatinib vs. TACE alone	Expected completion date in early 2027 with primary endpoint of PFS

## Data Availability

No new data were created or analyzed in this study.

## Abbreviations

The following abbreviations are used in this manuscript:

ALBI	Albumin–bilirubin
ART	Assessment for retreatment with TACE
BCLC	Barcelona Clinic Liver Cancer
B-TACE	Balloon-occluded transarterial chemoembolization
CI	Confidence interval
cTACE	Conventional transarterial chemoembolization
CTLA-4	Cytotoxic T-lymphocyte-associated protein 4
DEB-TACE	Drug-eluting bead transarterial chemoembolization
DFS	Disease-free survival
EBRT	Electron beam radiotherapy
ECOG	Eastern Cooperative Oncology Group
HBV	Hepatitis B virus
HCC	Hepatocellular carcinoma
HCV	Hepatitis C virus
HR	Hazard ratio
INR	International normalized ratio
MASLD	Metabolic dysfunction-associated steatotic liver disease
miRNAs	Micro-RNAs
MWA	Microwave ablation
NETs	Neutrophil extracellular traps
NLR	Neutrophil-to-lymphocyte ratio
OS	Overall survival
PD-1	Programmed cell death protein 1
PD-L1	Programmed cell death ligand 1
PFS	Progression-free survival
RCT	Randomized controlled trial
RFA	Radiofrequency ablation
RFS	Relapse-free survival
RILD	Radiation-induced liver disease
RR	Relative risk
RT	Radiation therapy
SBRT	Stereotactic body radiotherapy
SIRT	Selective internal radiotherapy
TAE	Transarterial embolization
TACE	Transarterial chemoembolization
TACE-RT	Transarterial chemoembolization with radiation therapy
TARE	Transarterial radioembolization
TTP	Time to progression
VEGF	Vascular endothelial growth factor
Y-90	Yttrium-90
